# Identification and validation of targets of swertiamarin on idiopathic pulmonary fibrosis through bioinformatics and molecular docking-based approach

**DOI:** 10.1186/s12906-023-04171-w

**Published:** 2023-10-05

**Authors:** Jun Chang, Shaoqing Zou, Yiwen Xiao, Du Zhu

**Affiliations:** College of Life Science, Jiangxi Science & Technology Normal University, Nanchang, Jiangxi China

**Keywords:** Idiopathic pulmonary fibrosis, Traditional Chinese medicine, Swertiamarin, Bioinformatics, Molecular docking

## Abstract

**Background:**

Swertiamarin is the main hepatoprotective component of *Swertiapatens* and has anti-inflammatory and antioxidation effects. Our previous study showed that it was a potent inhibitor of idiopathic pulmonary fibrosis (IPF) and can regulate the expressions of α-smooth muscle actin (α-SMA) and epithelial cadherin (E-cadherin), two markers of the TGF-β/Smad (transforming growth factor beta/suppressor of mothers against decapentaplegic family) signaling pathway. But its targets still need to be investigated. The main purpose of this study is to identify the targets of swertiamarin.

**Methods:**

GEO2R was used to analyze the differentially expressed genes (DEGs) of GSE10667, GSE110147, and GSE71351 datasets from the Gene Expression Omnibus (GEO) database. The DEGs were then enriched with Gene Ontology (GO) and Kyoto Encyclopedia of Genes and Genomes (KEGG) analysis for their biological functions and annotated terms. The protein-protein interaction (PPI) network was constructed to identify hub genes. The identified hub genes were predicted for their bindings to swertiamarin by molecular docking (MD) and validated by experiments.

**Results:**

76 upregulated and 27 downregulated DEGs were screened out. The DEGs were enriched in the biological function of cellular component (CC) and 7 cancer-related signaling pathways. Three hub genes, i.e., *LOX* (lysyl oxidase), *COL5A2* (collagen type V alpha 2 chain), and *CTGF* (connective tissue growth factor) were selected, virtually tested for the interactions with swertiamarin by MD, and validated by in vitro experiments.

**Conclusion:**

LOX, COL5A2, and CTGF were identified as the targets of swertiamarin on IPF.

**Supplementary Information:**

The online version contains supplementary material available at 10.1186/s12906-023-04171-w.

## Introduction

IPF is a chronic progressive disorder and shares many common clinical, pathological, and immune characteristics with pulmonary fibrosis [1], one of the post-sequelae of COVID-19 [2]. IPF can significantly lower patients’ life quality and life expectancy [3] because of the irreversible decline in lung functions. Some environmental factors, genetic susceptibilities [4], and oxidative stress [5] were reported to increase the risk of getting IPF. Oxidative stress is believed to play an important role in the initiation of inflammation and the damage to DNA [6], which first occurs in the development of IPF. Various signaling pathways, e.g., hedgehog [7], TGF-β/Smad [8], and Wnt/β-catenin (Wingless and Int-1/beta catenin) [9], etc., are involved in the initiation and development of the IPF. Marker proteins of these signaling pathways, for instance, TGFBRI (transforming-beta type I receptor) [10] and CTGF [11], etc., were reported to be the targets of anti-IPF drugs. Our previous study of screening novel anti-IPF drugs with machine learning from traditional Chinese medicines found that swertiamarin, a secoiridoid glycoside with high anti-oxidation and anti-inflammatory effects [12], has a strong effect on arresting the development of IPF [13], and identified that α-SMA and E-cadherin, two marker proteins of TGF-β/Smad signaling pathway, were significantly downregulated and upregulated, respectively, by swertiamarin. However, the targets still need to be investigated. For this new anti-IPF lead, it is tough and time-and-cost-consuming work to identify its targets with wet-lab experiments. Fortunately, bioinformatics provides us with powerful tools to exploit genomic, transcriptomic, and proteomic data [14] to gain insights into the anti-IPF mechanisms and to identify targets for further validation. Molecular docking (MD) is another effective *in silico* approach to accurately predict the stability of a ligand-receptor complex and understand the activity of the ligand. MD showed remarkable advantages [15] over traditional experiment paradigms in avoiding large amounts of intensive experiments. In this study, bioinformatics was first used to analyze the microarray datasets from the GEO database to obtain hub genes. MD was then applied to decide whether these hub genes were the targets of swertiamarin by predicting the interactions between swertiamarin and the corresponding proteins of these genes. Finally, the screened targets were experimentally validated.

## Materials and methods

### Microarray data

GSE10667 series [16] on GPL4133 platform (Agilent-014850 Whole Human Genome Microarray 4 × 44 K G4112F), GSE110147 series [17] on GPL6244 platform (Affymetrix Human Gene 1.0 ST Array transcript), and GSE71351 series [18] on GPL10558 platform (Illumina HumanHT-12 V4.0 expression bead chip) were downloaded from the GEO database (https://www.ncbi.nlm.nih.gov/geo/). The probs in each series were replaced by official gene symbols according to the platform files. For the GSE10667 dataset, the samples with the title of control and UIP (usual interstitial pneumonia) were set as control and IPF groups, respectively. The samples of the GSE110147 dataset that has the title of normal control and idiopathic pulmonary fibrosis patient were selected as control and IPF groups, respectively. In the GSE71351 dataset, the samples of normal lung fibroblasts were set as the control group and those of rapidly/slowly lung fibroblasts were set as the IPF group. The detailed information on these groups is shown in Table 1. The above three datasets are freely accessed on the GEO database and do not include any other experiment data of the authors.

**Table 1:** Statistics of the three microarray databases

Dataset	Control	IPF	Total number
GSE10667	15	23	38
GSE110147	11	22	33
GSE71351	4	8	12

### Identification of differentially expressed genes

To avoid the batch effect, GSE10667, GSE110147, and GSE71351 datasets were separately analyzed for DEGs with GEO2R (https://www.ncbi.nlm.nih.gov/geo/geo2r) between control and IPF groups. The genes with |logFC|≥0.5 and *P* ≤ 0.05 were identified as DEGs. The Venn Diagram (http://bioinformatics.psb.ugent.be/webtools/Venn/) was used to select the overlapping genes that simultaneously exist in the DEGs of GSE10667, GSE110147, and GSE71351 datasets for GO and KEGG enrichment analysis.

### GO and KEGG function enrichment analyses

The overlapping DEGs including upregulated and downregulated genes were used for GO and KEGG [19] enrichment analyses with David [20] (https://david.ncifcrf.gov/). For the GO analysis, the result was filtered with *P* ≤ 0.05 and Count ≥ 10 and the resultant genes were investigated for their classified physiological functions i.e., cellular component (CC), and their annotated terms. For KEGG analysis, the genes with *P* ≤ 0.05 and Count ≥ 4 were considered as significantly enriched in the corresponding biological pathways.

### PPI network and hub genes identification

The DEGs including upregulated and downregulated genes were used to construct PPI networks with STRING [21]. The PPI pairs were extracted with a combined score of 0.4. Nodes with higher degrees of connectivity are considered more important to the stability of the PPI network. The PPI networks were plotted by Cytoscape 3.9.1.

The hub genes were calculated with the CytoHubba [22] plugin of Cytoscape 3.9.1 [23] and the algorithm of *Closeness* was used to rank the nodes from which the top 3 hub genes were selected for validation.

### Molecular docking

To decide whether the selected hub genes were the potential targets of swertiamarin, MD was used to predict the interactions between swertiamarin and the proteins of selected genes. 3D crystal structure of human LOX (PDB ID 5ZE3) [24] with a resolution of 2.40Å was downloaded from the PDB database (https://rcsb.org/structure/5ZE3). The 3D structures of CTGF (https://alphafold.ebi.ac.uk/entry/P29279) and COL5A2 (https://alphafold.ebi.ac.uk/entry/P05997) were obtained from AlphaFold. The docking pocket of LOX was decided with its catalytic domain [24]. The docking pocket of CTGF was defined as its heparin-binding region (https://www.uniprot.org/uniprotkb/P29279/entry). The VWFC domain of COL5A2 protein (https://www.uniprot.org/uniprotkb/P05997/entry) was used as its docking pocket. Autodock vina 1.2.3 [25] was used to perform the MD of swertiamarin into LOX, CTGF, and COL5A2. The structural files of LOX, CTGF, COL5A2 in PDB format, and swertiamarin in SD format were converted into PDBQT format with OpenBabel 2.4.1 [26] for docking, and other docking parameters were set as default.

### Western blot testing

The western blot experiments were carried out with three experiment groups, i.e., the control, the IPF model, and the test groups (detailed information is shown in Fig. 5). The A549 cells from the above three groups were lysed with RIPA (radioimmunoprecipitation assay) lysis buffer and vortexed at 4℃. After the cells were collected. Total protein was loaded and separated by electrophoresis on 10% SDS-PAGE (sodium dodecyl sulfate-polyacrylamide gel electrophoresis) and transferred onto polyvinylidene difluoride membranes (Millipore, USA). The membranes were blocked with 5% defatted milk for 1.5 h at 20℃ and then incubated with primary antibodies at 4˚C overnight. After being washed with 10% TBST (mixture of tris-buffered saline and polysorbate 20), the membranes were incubated with HRP-conjugated (horseradish peroxidase-conjugated) secondary antibodies of anti-mouse IgG (immunoglobulin G) (1:5000) and anti-rabbit IgG (1:5000) for 2 h at 20℃. The bound antibodies were visualized using an enhanced chemiluminescence kit (Millipore, USA).

### Statistical analysis

The GEO analyses were carried out by the GEO2R. The statistical analysis of expressions of the hub genes in the western blot experiments was performed by Pearson’s correlation analysis in the Python environment. The confidence interval was set as 0.95 for DEGs and western blot analysis.

## Results

### Identification of DEGs

**Fig. 1:**
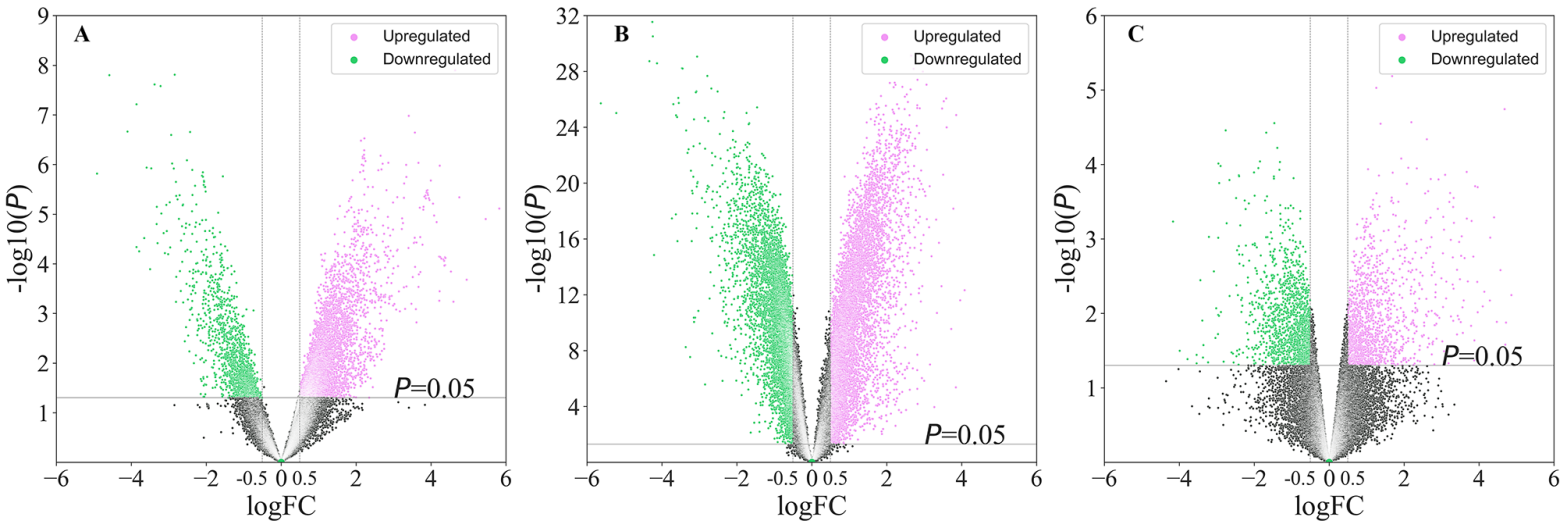
The distributions of gene expressions of GSE10667 (**A**), GSE110147 (**B**), and GSE71351 (**C**) datasets. The genes with log|FC|≥0.5 and *P* ≤ 0.05 were considered as DEGs.

**Fig. 2:**
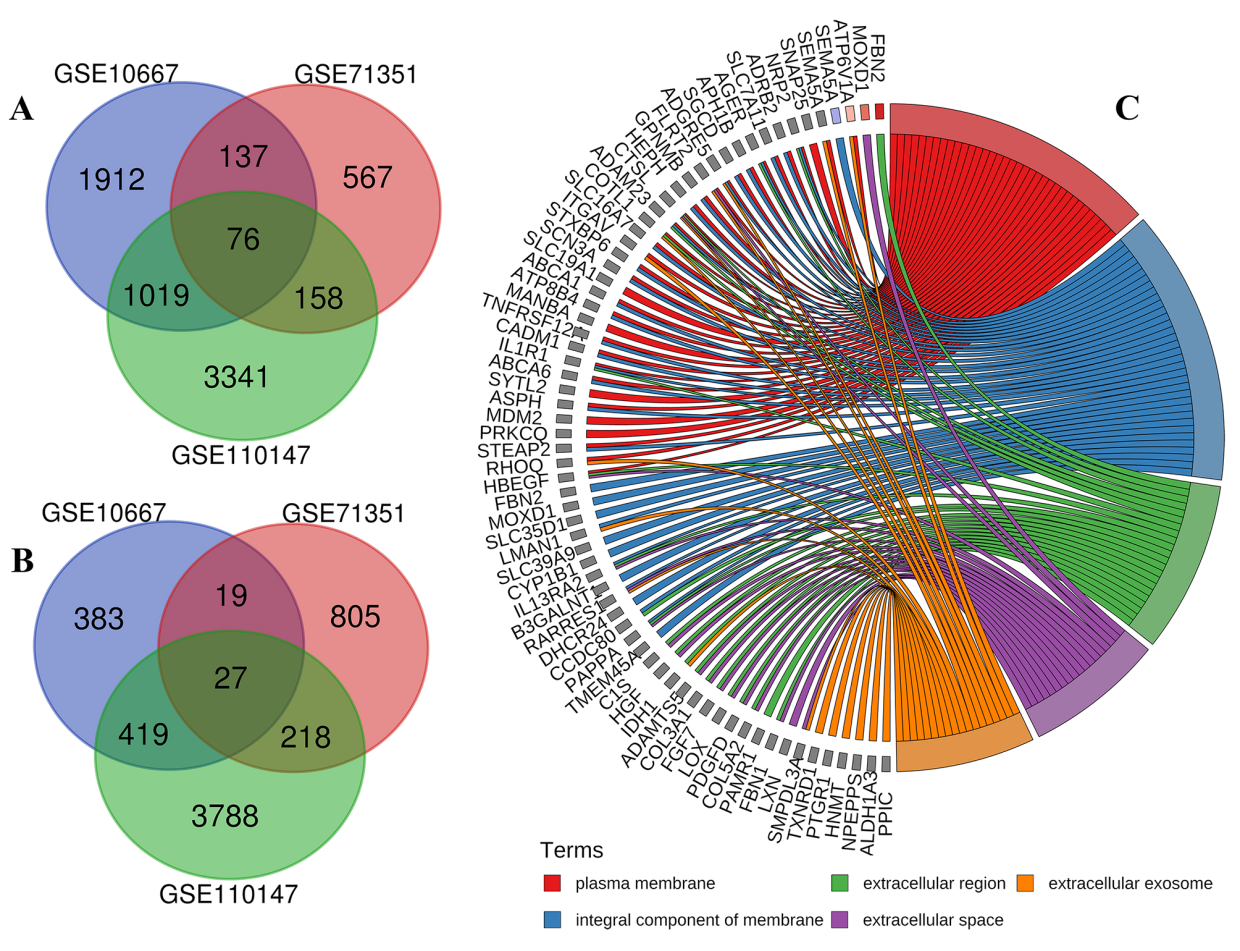
The DEGs and the biological functions of GO-enriched genes. **A**, the intersecting upregulated genes. There are 76 overlapping upregulated genes (supplement 1). **B**, the intersecting downregulated genes. There are 27 overlapping downregulated genes (supplement 2); **C**, the biological functions of DEGs enriched with GO analysis. The GO-enriched genes were selected with Count ≥ 18 and *P* ≤ 0.05

Three gene expression profiles (GSE10667, GSE110147, and GSE71351) were selected and analyzed. The GSE10667, GSE110147, and GSE71351 datasets contained 15 normal samples and 23 IPF (usual interstitial pneumonia) samples, 11 normal samples and 22 IPF (rapidly/slowly lung fibroblast) samples, and 4 normal samples and 8 IPF samples, respectively (Table 1). There are 3144, 4594, and 938 genes that were upregulated in GSE10667, GSE110147, and GSE71351 datasets (Fig. 1), respectively. And a total of 76 overlapping genes were found upregulated (Fig. 2A). There are 848, 4452, and 1069 genes that were downregulated in the GSE10667, GSE110147, and GSE71351 datasets (Fig. 1), respectively, from which 27 overlapping genes were observed (Fig. 2B). The DEGs were mainly related to the biological functions of the plasma membrane, extracellular region, extracellular exosome, integral component of membrane, and extracellular space (Fig. 2C).

.

### GO and KEGG enrichment analysis

The GO enrichment aims to analyze the main functions, e.g., cellular component, biological process, molecular function, etc., of DEGs. The results (Table 2) showed that the DEGs were enriched in cellular components including the plasma membrane, integral component of membrane, extracellular region, extracellular space, extracellular exosome, and Golgi apparatus, which were consistent with the biological process of IPF. The results of KEGG analysis indicated that these DEGs were mainly enriched in 7 pathways including Hsa05202, Hsa04020, Hsa04115, Hsa04068, Hsa04978, Hsa05218, and Hsa05205, which are relative to cancer and tissue development.

**Table 2:** Significantly enriched GO terms and KEGG pathways of DEGs

Biological functions	Term	Count	*P*
Cellular component	Plasma membrane	35	0.023
Cellular component	An integral component of the membrane	35	0.029
Cellular component	Extracellular region	22	0.000
Cellular component	Extracellular space	18	0.008
Cellular component	Extracellular exosome	18	0.027
Cellular component	Golgi apparatus	11	0.040
Transcriptional misregulation in cancer	Hsa05202	5	0.042
Calcium signaling pathway	Hsa04020	6	0.023
p53 signaling pathway	Hsa04115	4	0.013
FoxO signaling pathway	Hsa04068	5	0.012
Mineral absorption	Hsa04978	4	0.008
Melanoma	Hsa05218	5	0.001
Proteoglycans in cancer	Hsa05205	5	0.048

### PPI network and hub genes

**Fig. 3:**
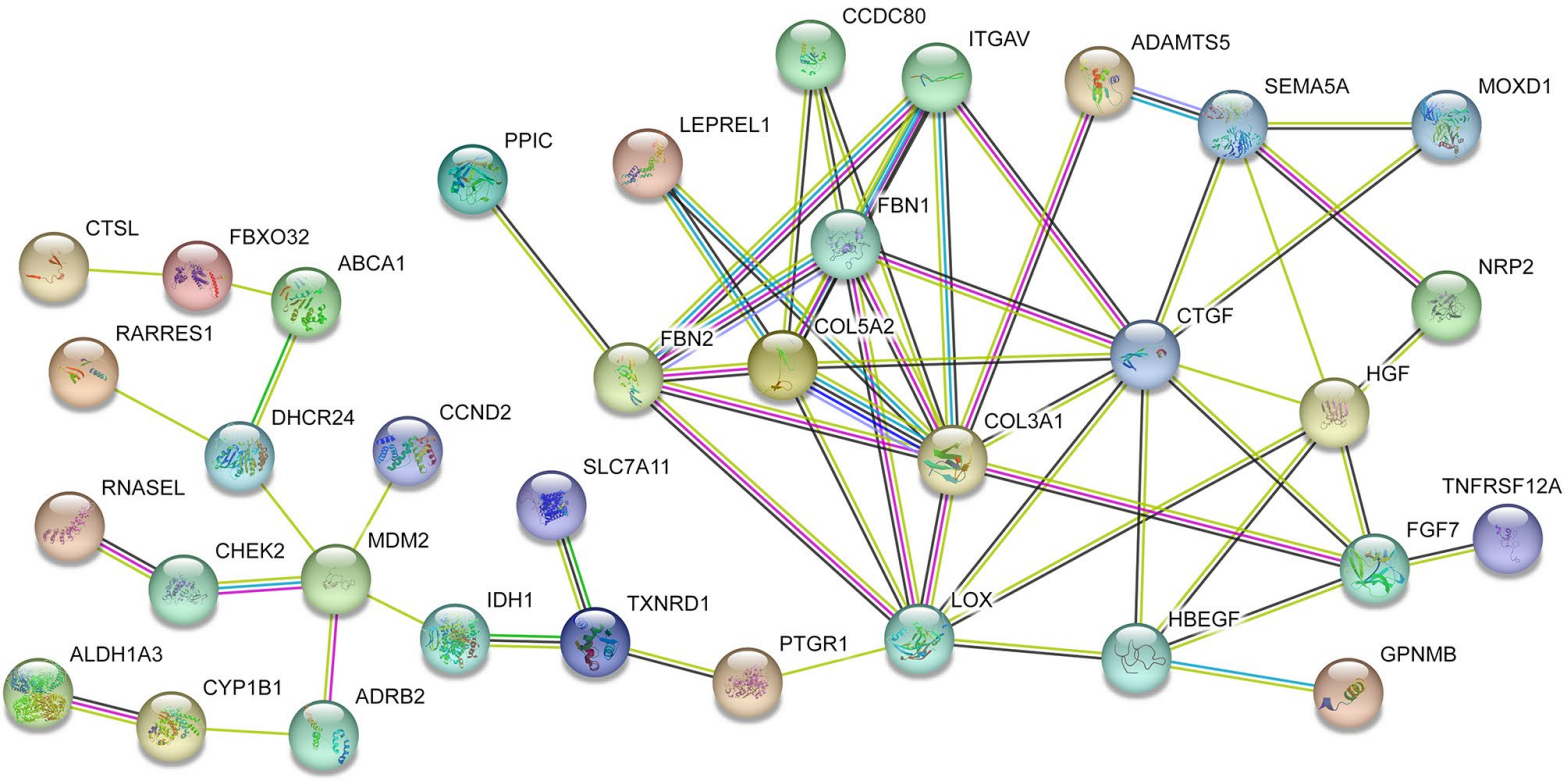
The PPI network of DEGs.

The protein interactions among the DEGs (supplement 3) were constructed with STRING and filtered by confidence over 0.4. The results showed that a total of 50 nodes and 130 edges were involved in the PPI network (Fig. 3). There are 12 nodes i.e., *LOX*, *COL5A2*, *COL3A1* (collagen type III alpha 1), *FNIP1* (folliculin interacting protein 1), *FBN2* (fibrillin 2), *ADAMTS5* (ADAM metallopeptidase with thrombospondin type 1 motif 5), *SEMA5A* (semaphorin 5 A), *FBN1* (fibrillin 1), *ATP6V1A* (ATPase H + transporting V1 subunit A), *ITGAV* (integrin subunit alpha V), *CHEK2* (checkpoint kinase 2), and *MDM2* (MDM2 proto-oncogene) and 122 edges have combined scores over 0.85. The result of hub genes calculation (Table 3) showed that *CTGF* and *COL5A2* were the two most outstanding genes with scores of 20, followed by *LOX* (score = 19), *COL3A1* (score = 16), *FBN1* (score = 14), *HGF* (hepatocyte growth factor, score = 12), *FBN2* (score = 12), *HBEGF* (heparin-binding EGF like growth factor, score = 10), *FGF7* (fibroblast growth factor 7, score = 10) and *ITGAV* (score = 10). Finally, the top 3 genes (*CTGF*, *COL5A2*, and *LOX*) were selected for the validation.

**Table 3:** The top ten hub genes ranked with scores

Gene names	Gene descriptions	Scores
*CTGF*	Connective tissue growth factor	20
*COL5A2*	Collagen type V alpha 2 chain	20
*LOX*	Lysyl oxidase	19
*COL3A1*	Collagen type III alpha 1 chain	16
*FBN1*	Fibrillin 1	14
*HGF*	Hepatocyte growth factor	12
*FBN2*	Fibrillin 2	12
*HBEGF*	Heparin-binding EGF-like growth factor	10
*FGF7*	Fibroblast growth factor 7	10
*ITGAV*	Integrin subunit alpha V	10

### Molecular docking

**Fig. 4:**
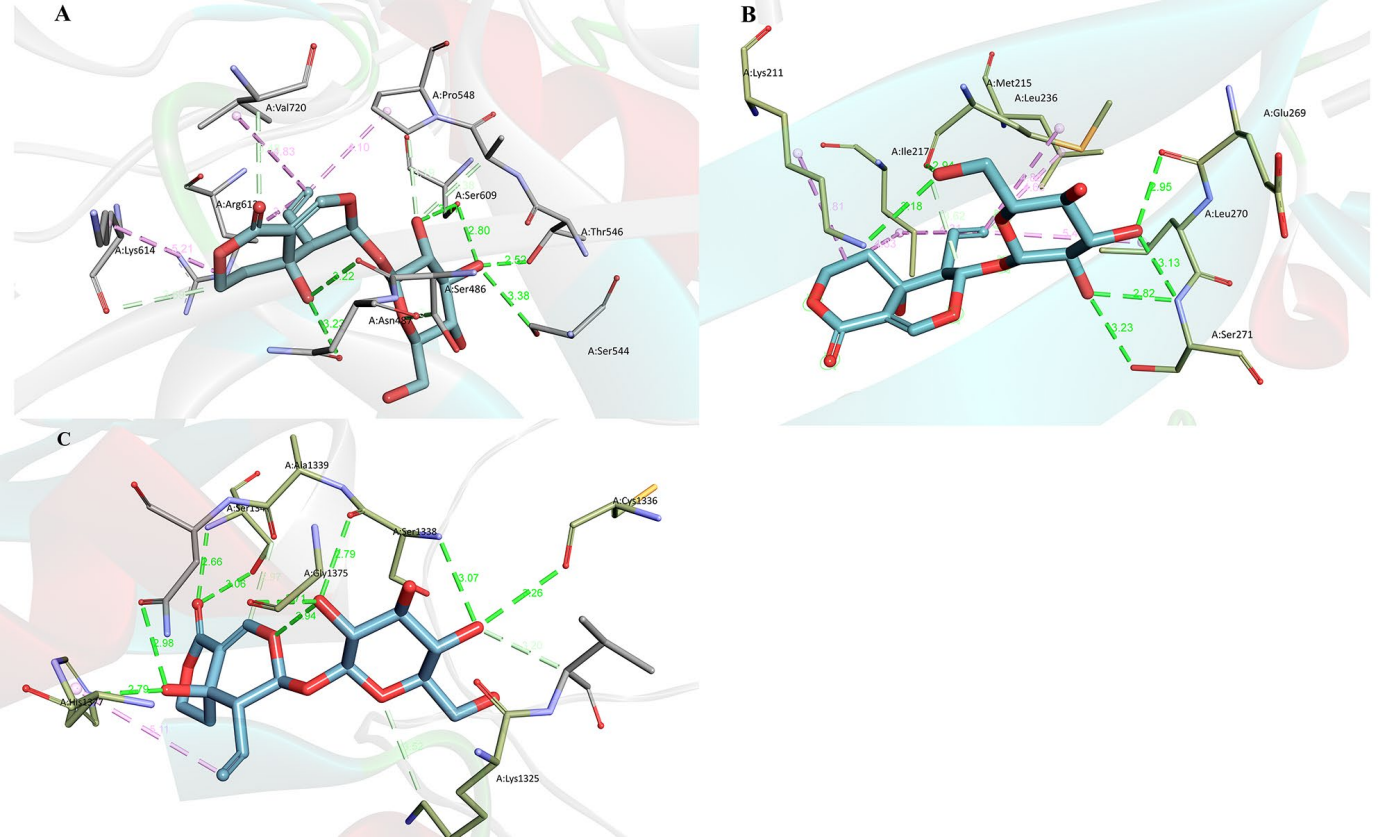
The 3D interactions between swertiamarin and LOX (**A**), CTGF (**B**), and COL5A2 (**C**), respectively. Green lines, conventional hydrogen bonds. Pink lines, alkyl bonds. Light blue lines, conventional hydrogen bonds. The numbers beside the bonds represent the bond lengths. The interactions were plotted by the Discovery Studio 2019 client

The docking score for swertiamarin and LOX was − 9.344 kcal/mol and the interactions were shown in Fig. 4A. Besides 5 carbon-hydrogen bonds, 6 conventional hydrogen bonds (between swertiamarin and residues of Ser609, Thr546, Ser544, Ser486, and Asn487 of LOX with bond lengths of 3.11, 2.80, 2.52, 3.38, 3.22 and 3.23Å, respectively) and 3 alkyl bonds (between swertiamarin and residues of Pro548, Val720, and Arg612 of LOX with bond lengths of 4.10, 4.83 and 3.68Å, respectively) were found in the swertiamarin-LOX complex, which indicated that swertiamarin has strong interactions with LOX and the swertiamarin-LOX complex was very stable.

The docking score for swertiamarin and CTGF was − 8.125 kcal/mol. The swertiamarin-CTGF interactions (Fig. 4B) gave out 5 conventional hydrogen bonds (between swertiamarin and Glu269, Ser271, Met215, and Lys211 with bond lengths of 2.95, 3.13, 3.57, 2.94, and 3.18Å, respectively) and 6 alkyl bonds (between swertiamarin and Met215, Leu236, Len270, Ile217, and Lys21 with bond lengths of 4.86, 4.66, 5.42, 4.21, 4.83 and 3.81Å, respectively). The vinyl of the swertiamarin formed 3 alkyl bonds with residues of Leu270, Ile217, and Leu236, which suggested that this vinyl is important to the stability of the complex. The residue of Ser271 provided three strong conventional hydrogen bonds (bond lengths < 3Å) with swertiamarin, which suggested that the Ser271 and the hydroxyls from the glucosyl of swertiamarin are the key groups keeping the stability of the swertiamarin-CTGF complex.

The docking score for swertiamarin and COL5A2 was − 12.681 kcal/mol, which indicated that the swertiamarin-COL5A2 complex is more stable than the swertiamarin-LOX and swertiamarin-CTGF complexes. The swertiamarin-COL5A2 interactions (Fig. 4C) showed the same result. There were more and stronger conventional hydrogen bonds were formed: six of the nine conventional hydrogen bonds are shorter than 3Å and three conventional hydrogen bonds are close to 3Å (3.26Å between swertiamarin and Cys1336, 3.07Å between swertiamarin and Ser1338, and 3.06Å between swertiamarin and Ser1342). Therefore, glucosyl is very important to the anti-IPF capability of swertiamarin.

### Western blot

**Fig. 5:**
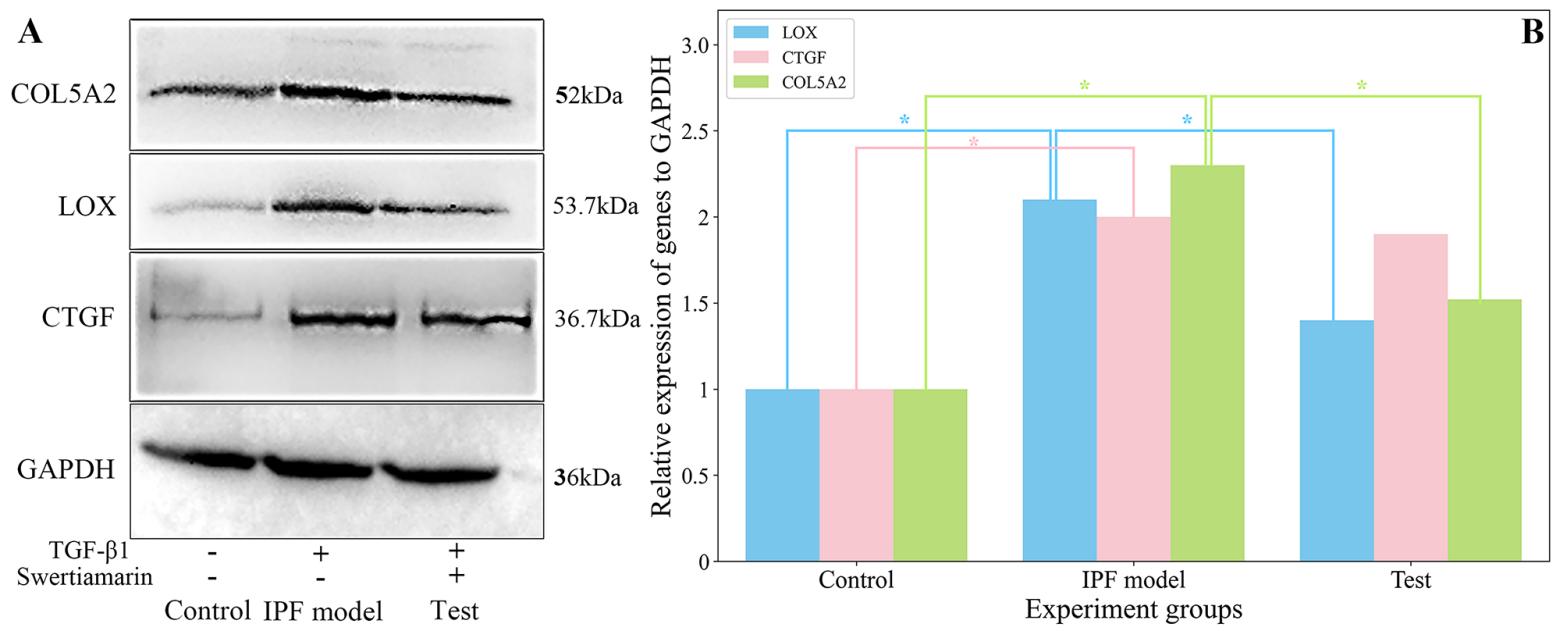
The expressions (**A**) and the statistics results (**B**) of *COL5A2*, *LOX*, and *CTGF* in the western blot analysis. *Represents the *P* < 0.05. The A549 cells (control group) were pretreated with 10ng/ml of TGF-β1 to build the in vitro IPF model, and then the cells were treated with 1.5µmol/l of swertiamarin (test group) for 24 h. The samples for determining the expressions of *COL5A2*, *LOX*, *CTGF*, and *GAPDH* (glyceraldehyde 3-phosphate dehydrogenase) were derived from the same batch of experiments. The gels were processed in parallel

Experiment results showed that TGF-β1 and swertiamarin have no toxicity to the A540 cells (supplement 4). Figure 5 showed that, after being treated with swertiamarin for 24 h, the expressions of *LOX* and *COL5A2* were significantly downregulated, and the expression of *CTGF* was observed to be slightly downregulated.

## Discussion

Target identification is of central importance to the understanding of the anti-IPF mechanism of swertiamarin. However, solving this problem using web-lab experiments usually means expensive and slow processes, whereas computation-aided approaches provide efficient complements. Here we used the bioinformatics and MD-based approach to screen the anti-IPF targets of swertiamarin with the public database. In this study, GSE10667, GSE110147, and GSE71351 datasets were used to analyze the DEGs. It is important to clarify here that the GSE10667 dataset contains samples with interstitial pneumonia and it made the results more reliable but also more difficult to obtain the overlapping DEGs with high log|FC| values because of considering the gene expressions in the early stage of IPF. Even with lower log|FC| values, the hub genes were still successfully screened out. The KEGG analysis suggested that the selected DEGs were related to tissue and cancer development. This result was consistent with the fact that IPF shares several pathogenetic similarities, e.g., DNA methylation [27], with lung cancer [28] and that patients with IPF are at high risk of getting lung cancer [29].

Three hub genes (*LOX*, *COL5A2*, and *CTGF*) were screened out through the above analysis. The LOX is a cuproenzyme and is also known as protein-lysine 6-oxidase encoded by the human *LOX* gene [30]. LOX catalyzes the conversion of lysine into highly reactive aldehydes that form the cross-linking collagen and elastin in ECM proteins [31], contributing to the ECM’s stiffness. The stiffness of the ECM would increase fibroblast proliferation and contraction [32]. Aberrant expression and activity of LOX were associated with IPF [33] and led to the development of the IPF microenvironment. Therefore, LOX is a key participant in ECM remodeling [34]. In this study, the PPI analysis of LOX showed that the LOX has strong interactions with TGF-β1, TGFBR1, TGFBR2 (TGF-β receptor 2), and SMAD2/3 (suppressor of mothers against decapentaplegic family member 2 and 3) (Supplement 5), which are the main marker proteins of TGF-β/Smad signaling pathway. Therefore, LOX is the potential target of IPF [35] and inhibition of its activity would alleviate the IPF [33], which was identified by the MD model. The interactions between LOX and swertiamarin indicated a stable ligand-receptor complex (Fig. 4A), which is consistent with the western blot results showing the downregulation of the *LOX* expression.

*CTGF*, also known as *CCN2* (cellular communication network factor 2), is a matricellular protein of the *CCN* family of ECM-associated heparin-binding proteins [36]. CTGF was reported to be associated with wound healing (the key initial process of IPF) and all fibrotic pathology [37]. In IPF tissues, *CTGF* expression is upregulated by TGF-βs [38], SMAD2 [39], and other physiological and pathological factors. The upregulation of *CTGF* expression would further exacerbate the ECM accumulation and aggravate the development of IPF [37]. The CTGF-involved PPI analysis (Supplement 6) showed that CTGF has interactions with TGF-β1, TGF-β3, and TGFBR2 (one of the key targets of exogenous factors that promote the expression of TGF-βs). The MD model also suggested that the swertiamarin-CTGF complex is highly stable and CTGF should be the target of swertiamarin on IPF. Western blot results showed that swertiamarin slightly but not significantly downregulated the expression of CTGF. Therefore, we concluded that swertiamarin can only inhibit the activity of CTGF rather than downregulate the expression of *CTGF*.

COL5A2 is a protein encoded by the *COL5A2* gene [40] and is responsible for the formation of other collagen fibrils in tissues of the body. COL5A2 was reported to be involved in the development of pathological scarring [41]. *COL5A2* is modulated by TGF-βs [42] and is highly related to human systemic sclerosis [43] and IPF [44]. In this study, the COL5A2 protein has a strong interaction with ITGB1 (integrin beta-1, a cell surface receptor in human and function as collagen receptors and modulate the migration across basement membranes in human [45]) and ADAMTS14 (ADAM metallopeptidase with thrombospondin type 1 motif 14, an enzyme cleaves the amino-propeptide of fibrillar collagens) (Supplement 7). The DEG analysis showed that the *COL5A2* gene was significantly upregulated in the IPF patients. The MD model suggested that swertiamarin could modulate the activity of COL5A2 and the western blot showed that the expression of *COL5A2* was downregulated by swertiamarin.

## Conclusion

Our bioinformatics analysis identified 103 DEGs that co-exist in GSE10667, GSE110147, and GSE71351 datasets. The GO and KEGG functional analyses showed that these DEGs were mainly related to the cell process that related to cancer and tissue development. The MD models and experimental results proved that these three genes/proteins are the targets of swertiamarin. The experiment data didn’t show significant downregulation of *CTGF*. It is very important to further experimentally investigate the effects of swertiamarin on the activities of LOX, CTGF, and COL5A2, and especially to validate its effect on the expression of *CTGF*.

### Electronic supplementary material

Below is the link to the electronic supplementary material.

Supplementary Material 1

Supplementary Material 2

Supplementary Material 3

Supplementary Material 4

Supplementary Material 5

Supplementary Material 6

Supplementary Material 7

## Data Availability

GSE10667, https://www.ncbi.nlm.nih.gov/geo/query/acc.cgi?acc=GSE10667. GSE110147, https://www.ncbi.nlm.nih.gov/geo/query/acc.cgi?acc=GSE110147. GSE71351, https://www.ncbi.nlm.nih.gov/geo/query/acc.cgi?acc=GSE71351.

## List of Abbreviations

IPF: Idiopathic pulmonary fibrosis
GEO: Gene Expression Omnibus
UIP: Usual interstitial pneumonia
DEG: Differentially expressed genes
CC: Cellular component
GO: Gene Ontology
KEGG: Kyoto Encyclopedia of Genes and Genomes
PPI: Protein-protein interaction network
TGF-β: Transforming growth factor beta
TGFBRI: Transforming-beta type I receptor
LOX: Lysyl oxidase
COL5A2: Collagen type V alpha 2 chain
CTGF: Connective tissue growth factor
CCN2: Cellular communication network factor 2
SMAD2: suppressor of mothers against decapentaplegic family member 2
SMAD3: Suppressor of mothers against decapentaplegic family member 3
GAPDH: Glyceraldehyde 3-phosphate dehydrogenase.
ADAMTS14: ADAM metallopeptidase with thrombospondin type 1 motif 14
α-SMA: α-smooth muscle actin
E-cadherin: Epithelial cadherin
ECM: Extracellular matrix
Wnt: Wingless and Int-1
MD: Molecular docking
TBST: Mixture of tris-buffered saline and polysorbate 20
HRP: Horseradish peroxidase
RIPA: Radioimmunoprecipitation assay
