# Correction: Identification and validation of targets of swertiamarin on idiopathic pulmonary fibrosis through bioinformatics and molecular docking-based approach

**DOI:** 10.1186/s12906-024-04616-w

**Published:** 2024-08-21

**Authors:** Jun Chang, Shaoqing Zou, Yiwen Xiao, Du Zhu

**Affiliations:** College of Life Science, Jiangxi Science & Technology Normal University, Nanchang, Jiangxi China

**Correction** **:** **BMC Complement Med Ther 23, 352 (2023)**

**https://doi.org/10.1186/s12906-023-04171-w**

A correction has been made to Fig. 5. The sub-image of GAPDH (the last western blot image of Fig. 5A) was replicated and incorrectly uploaded instead of the sub-image for COL5A2 (the first line of the Fig. 5A). This error does not affect the conclusion of the publication.

**Fig. 5:**
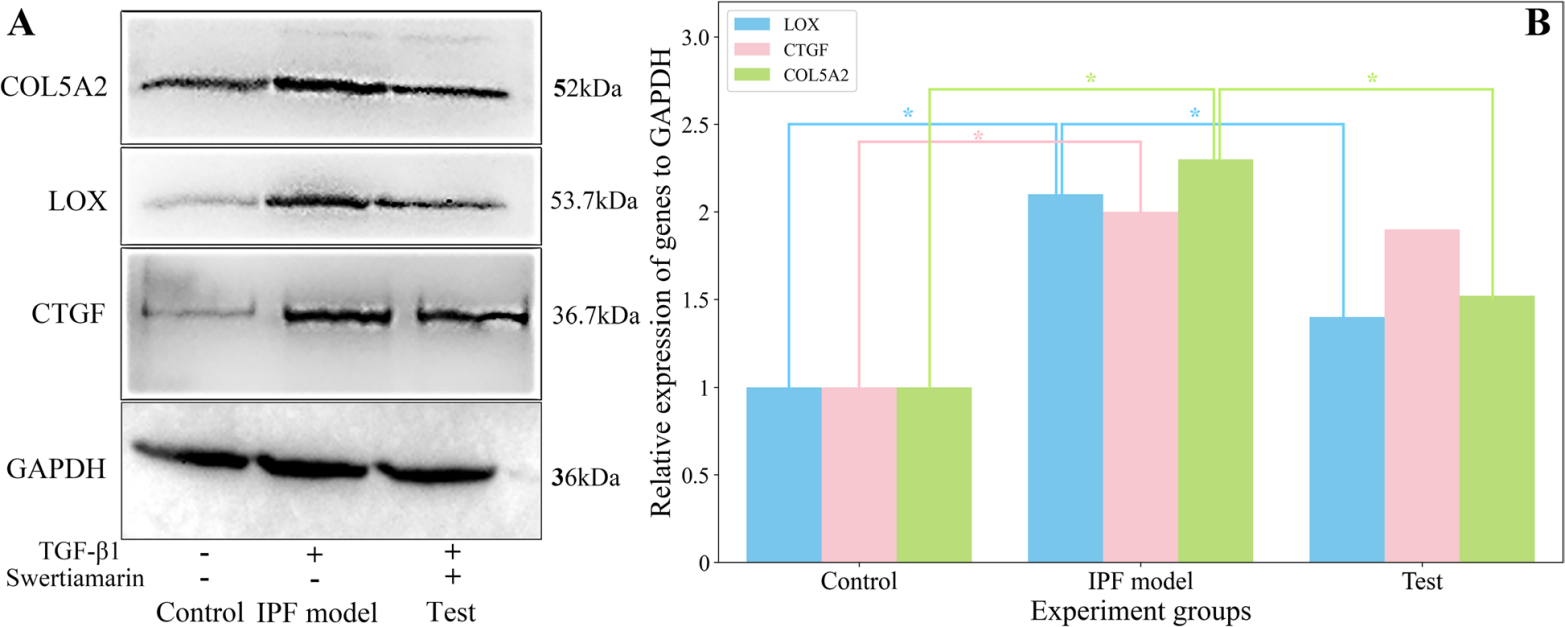
The expressions (**A**) and the statistics results (**B**) of COL5A2, LOX, and CTGF in the western blot analysis. *Represents the P < 0.05. The A549 cells (control group) were pretreated with 10ng/ml of TGF-β1 to build the in vitro IPF model, and then the cells were treated with 1.5μmol/l of swertiamarin (test group) for 24 h. The samples for determining the expressions of COL5A2, LOX, CTGF, and GAPDH (glyceraldehyde 3-phosphate dehydrogenase) were derived from the same batch of experiments. The gels were processed in parallel

The original article [1] has been corrected.
