# Supplementary material for: Identification and validation of targets of swertiamarin on idiopathic pulmonary fibrosis through bioinformatics and molecular docking-based approach
Source: BMC Complement Med Ther. 2023 Oct 5;23:352. doi: 10.1186/s12906-023-04171-w (PMC10557187; doi:10.1186/s12906-023-04171-w)
Supplement: Supplementary file 4 — Supplementary Material 4 [file 12906_2023_4171_MOESM4_ESM.pdf]

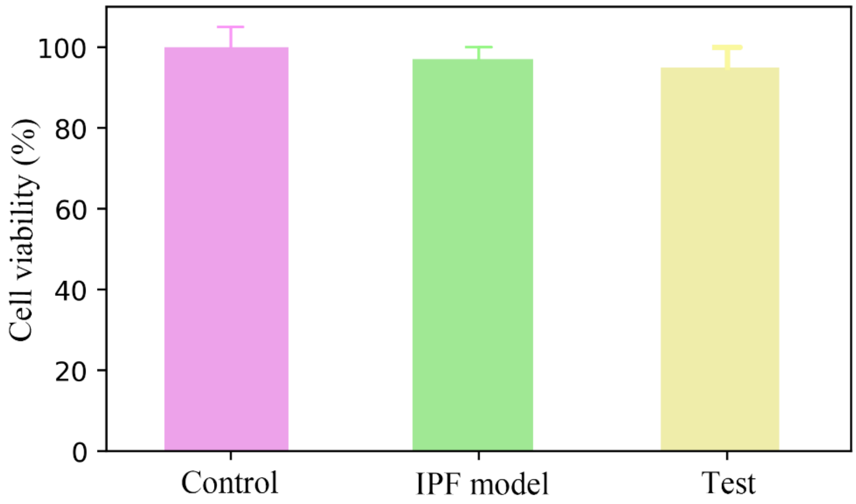

Supplement 4. The effect of swertiamarin on the viability of A549 cells.

The A549 cells with a concentration of 2000 cells/ml (control group) were induced by 10ng/ml of TGF-β1 (IPF model group) to build the *in vitro* IPF model. After 6h of culture, 1.5μmol/l of swertiamarin was added to the culture (test group). The cells were continued to be cultured under the same conditions for another 24h and then 10μl of CCK8 was added into each group. After being cultured for the next 4h, the OD_{450} values were measured to calculate the viabilities of cells for all groups.
