# Supplementary material for: Identification and validation of targets of swertiamarin on idiopathic pulmonary fibrosis through bioinformatics and molecular docking-based approach
Source: BMC Complement Med Ther. 2023 Oct 5;23:352. doi: 10.1186/s12906-023-04171-w (PMC10557187; doi:10.1186/s12906-023-04171-w)
Supplement: Supplementary file 7 — Supplementary Material 7 [file 12906_2023_4171_MOESM7_ESM.docx]

**
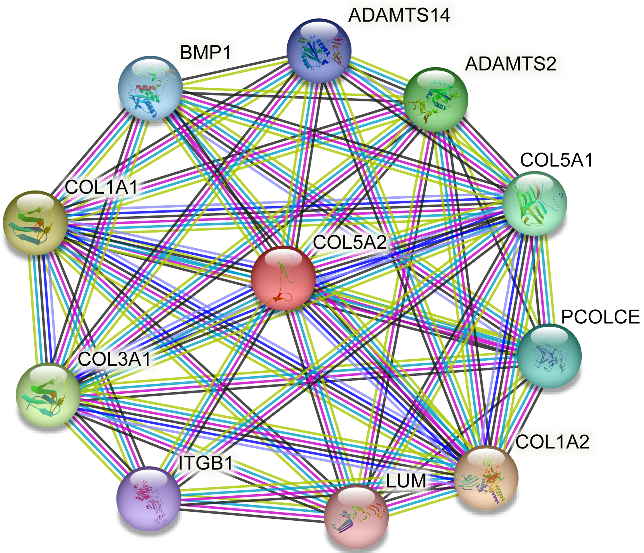
**

Supplement 7. The COL5A2 involved protein-protein network

The PPI network was construed with STRING and filtered by confidence over 0.4.
